# Who is more likely to start a business? Analysis of the factors influencing undergraduates’ entrepreneurial intentions

**DOI:** 10.3389/fpsyg.2022.829955

**Published:** 2022-08-23

**Authors:** Yan Yan Li, Ruo Xiang Wang, Chun Yang Chi

**Affiliations:** ^1^College of Education, Zhejiang University, Hangzhou, China; ^2^Development Planning Division, Wenzhou Vocational College of Science & Technology, Wenzhou, China; ^3^Party Committee & General Office, Wenzhou Polytechnic, Wenzhou, China; ^4^Editorial Department of Journal, Wenzhou Polytechnic, Wenzhou, China

**Keywords:** entrepreneurial intention, entrepreneurial education, higher education, influencing factors, entrepreneurial abilities

## Abstract

Entrepreneurial intention is an important condition for promoting substantive entrepreneurial behavior, which in turn alleviates employment pressure and promotes sustainable economic and social development. Based on national survey data of university students, descriptive statistical analysis of variables and binary logistic regression analysis were used to empirically analyze the factors influencing university students’ entrepreneurial intentions in five aspects, including innate endowment, acquired characteristics, entrepreneurship courses, and entrepreneurial practice, and social support. The regression results showed that both the factors of innate endowment and acquired characteristics passed the significance test. The entrepreneurship course factor had a significant impact on entrepreneurial intention, with the mean value of the number of entrepreneurship courses hitting 1.80, which is much lower than the other subdivision factors, indicating that there is a greater demand for entrepreneurship courses offered by university students, while the actual situation is less than satisfactory. The entrepreneurial practice factor is also an important factor, with an overall mean of 3 or more, and has a significant impact on entrepreneurial intentions, but in comparison, the factor of having an off-campus practice base for entrepreneurial practice has the lowest mean score, which shows that there is some room for improvement in the construction of off-campus practice bases. The social support factor significantly influences the willingness to start a business, and the mean score for each factor is above the medium level. By understanding which students are more willing to start a business and which factors are more likely to influence their willingness to start a business, we can further consider what kind of entrepreneurial skills and entrepreneurship education can lead to high-quality entrepreneurship and employment.

## Introduction

The number of university graduates in China has been increasing year by year since 1998, and has risen nearly 10-fold since then, and is still on the rise (Mycos, 2019). In the face of the increasing number of graduates, the employment situation of Chinese university students is complex and severe, and the difficulty of finding a good job is undoubtedly increasing. As individuals who can make rational choices, they will make judgments about the employment situation in the face of severe employment and thus adjust their employment expectations and employment needs accordingly (Li, 2020). Perhaps self-employment is a good “employment path” (Qu and Feng, 2019). China hopes to encourage more and more university students to engage in entrepreneurship through a deepening of the soft and hard environments. However, the willingness of Chinese university graduates to start their own businesses is generally low, with most choosing to continue their studies or find more stable jobs rather than start their businesses. Relevant findings indicate that the failure rate of entrepreneurship is as high as 70% globally, and the failure rate of entrepreneurship among Chinese university students is even higher than 90%, but less than 15% of these university entrepreneurs choose to start their business again (Liao et al., 2018

Entrepreneurship is a motivated behavior (Chen and Shi, 2020) and is not facilitated by entrepreneurial business opportunities, but rather by the entrepreneur’s willingness to start a business (Krueger, 2007). The concept of entrepreneurial will was first introduced by Bird (1988) and specifically refers to the state of consciousness in which an individual wants to create a new business. Entrepreneurial will is a psychological prerequisite for entrepreneurial action (Kolvereid and Moen, 1997; Noel, 2001) and without entrepreneurial will there is no entrepreneurial behavior. Only potential entrepreneurs with considerable entrepreneurial intentions are truly likely to engage in entrepreneurial activity (Krueger, 1993). Therefore, the entrepreneurial intention is one of the best predictors of entrepreneurial behavior (Krueger and Brazeal, 1994; Krueger, 2000) and a prerequisite for entrepreneurial success (Hasan et al., 2017). In turn, the formation of entrepreneurial intentions is a product of the interaction between the individual and the environment (Sun et al., 2011). Scholars have explored various factors from the external environment (Kristiansen and Indarti, 2004; Choo and Wong, 2006; Gurol and Atsan, 2006; Kuratko, 2014) or personal traits (Gartner, 1992; Baum and Singh, 1994; Simon et al., 2000; Zhao et al., 2005) to explore various factors that may contribute to entrepreneurial intentions. Some scholars have also recognized the inadequate explanatory power of environmental or individual-level variables for entrepreneurial behavior and have drawn on both the Theory of Planned Behavior model (Ajzen, 1985) and the Entrepreneurial Events model to discuss specifically the key factors influencing entrepreneurial intentions (Krueger et al., 2000; Hansen et al., 2004; Gelderen et al., 2008; Moriano et al., 2012; Yang, 2013; Maes et al., 2014; Xu et al., 2016; Feola et al., 2017). After understanding who is more willing to start a business, it is only then that the proportion of this group can be tapped and increased in a targeted manner, and precise support can be given to them so that they can move from having entrepreneurial intentions to substantive entrepreneurial behavior, effectively changing the low proportion of Chinese university students who start a business overall.

## Research review and research hypothesis

### Innate endowment

#### Gender

Research shows that gender-specific entrepreneurs are successful in different fields. In Austria, women are more likely to be successful in business and crafts entrepreneurship, while men specialize in information and consulting (Bögenhold and Klinglmair, 2017). Indeed, as early as the 1990s, the topic of gender-differentiated entrepreneurs, especially women entrepreneurs, gained widespread attention in Western societies due to the latter’s extremely rapid global growth rate (Brush et al., 2009), to be specific, female entrepreneurs in the United States grew at a rate of 9.4 between 1977 and 1984, which was 5.1 percentage points more than male entrepreneurs In the United States, female entrepreneurs grew at a rate of 9.4 between 1977 and 1984, 5.1 percentage points more than male entrepreneurs (Hisrich and Fan, 1991), and in Germany, there was a spurt in female entrepreneurs from 1991 to 2009, at 188.2% (Bögenhold and Fachinger, 2016), with academic research focusing on the background, motivations, Aparicio et al. (2021) show that women entrepreneurs benefit more from social mobility than men. Yet, rich research indicated that females express less entrepreneurial intentions due to certain underprivileged circumstances. Díaz-García and Jiménez-Moreno (2010) stated that in an extremely small number of samples, females emerging as entrepreneurs were discovered to receive less social acceptance in their immediate society, based on stereotypes of women and related consequences in terms of successfully assuming responsibility in business. Alsos and Ljunggren (2016) argued that females encountered certain structural barriers and stereotypical ascriptions when they operate the business. To be specific, Antoncic et al. (2013) found that different personality traits such as openness, extraversion, and agreeableness will affect entrepreneurial intentions when comparing the personality traits of male and female entrepreneurs. Meanwhile, Shinnar et al. (2012) examined challenges Chinese women entrepreneurs faced by applying cultural dimensions of Hofstede, turned out that female entrepreneurship in China perceived the lack of support as a strong on entrepreneurial intentions as Chinese men, even though they were not affected by the fear of failure and lack of competence. Furthermore, Plant and Ren (2010) confirmed that in China, as compared with the higher entrepreneurial rate of male students, the female gender was likely a negative factor for female students in terms of starting their businesses.

#### Ethnicity

Ethnic minority students, with their unique ethnic culture and special cultural resources, have some advantages in starting their businesses, as they can make use of their ethnic minority characteristics to attract market capital, as well as their own culture to the outside world, especially when they are engaged in types of entrepreneurships such as cultural creation. In the case of Chinese minority university students, “lack of experience” and “lack of capital” are often cited as two major obstacles to entrepreneurship (Chen, 2018). Differences in culture and values, living habits, and language expression between ethnic minorities and Han Chinese make ethnic minority students more likely to seek like-minded entrepreneurial partners among their peers and to receive support and assistance from capitalists of the same ethnic group (Tang et al., 2020).

#### One-child

The tendency in the Chinese and foreign literature to be lopsided as a “Chinese specific” factor, whether one child or not, is only mentioned by foreign scholars when they focus on a particular area, but the descriptions remain vague. Shinnar et al. (2012) stated that Chinese young people received “gender-neutral parental” care, which resulted in stronger confidence in competing with failure and a lack of capabilities to start a business, compared to those in America and Belgium. However, Pang (2016) claimed in a critical opinion that in fact, the greater part of children in China are “the only child” and have been completing their studies according to their parents’ design since childhood. Being highly dependent on parents, they lack the autonomy to engage in entrepreneurial struggle. After graduating, they will tend to look for jobs by their parents’ plans for them. Shen et al. (2022) studied the impact of entrepreneurial background on entrepreneurship and found that college students with entrepreneurial experience personally or with parents bear a higher entrepreneurial intention.

***Hypothesis 1: There is a significant positive effect of innate endowment on entrepreneurial intentions***

### Acquired characteristics

#### Type of household registration

As a factor related to the Chinese context, the impact of “hukou” (the Chinese type of household registration) on entrepreneurial intentions has attracted more general scholarly attention. In terms of urban and rural backgrounds, the entrepreneurial intentions of rural students are higher than those of urban students’ higher willingness to start a business than urban students (Liang and Shen, 2022). To be specific, it has been suggested that the type of household registration factor has a positive effect on the willingness to create a job. The desire to change the family situation is stronger among students with the rural residences. In the early stage of entrepreneurship, because of the large amount of human and material resources required, rural college students are more down-to-earth and hardworking under the guidance of national policies, so they will be more inclined to start a business when choosing employment or entrepreneurship, and nowadays, enterprises are more inclined to hire urban college students whose household registration is in the city where the enterprise is located, and rural college students have less obvious advantages in applying for jobs, which also increases the possibility of starting a business (Jiang, 2015).

#### Academic disciplines

Some scholars have studied the relationship between subject discipline and entrepreneurial intentions (Van de Ven et al., 1984; Salisu, 2020). Discipline categories were found to have the most substantial impact on entrepreneurial intentions (Ning and Ge, 2017; Çera et al., 2020).

There are differences in the willingness of graduates from different disciplines to consider starting their own business, as students from different disciplines will first consider the possibility of starting their own business. The study found that the group of entrepreneurial graduates was more from liberal arts majors (Min et al., 2006). However, some scholars believe that entrepreneurial intention is only related to the form of entrepreneurship education or entrepreneurial practice organization in their majors, and the richer the form of organization, the higher their involvement in entrepreneurship education in school, and the stronger their entrepreneurial intention. Since the fact that students have comprehensively learned entrepreneurship comprehension through an education system featuring entrepreneurship, thus obtaining inspiration and even resources for start-ups, the entrepreneurial intention of students majoring in entrepreneurship proves higher than that of students in other majors (Ruskovaara and Pihkala, 2014).

#### Understanding and skills

Bird (1995) held that personal traits, understanding, and skills constitute three key necessary components of entrepreneurship, which could be inborn or nurtured using education. According to Lee et al. (2006), entrepreneurship-related understanding and skills were requisite for entrepreneurial intentions. Originating in the United States, entrepreneurship education has aimed at cultivating students’ entrepreneurial consciousness and desire, reinforcing personal skills, and thus improving students’ entrepreneurial ability (Peterman and Kennedy, 2003). Indeed, emotional skills are also a very important and influential part of the entrepreneurial journey and need to be fully mastered for the challenging entrepreneurial journey (Aly et al., 2021).

#### Social resources

Students have very limited social capital and if their social capital is large, they are more likely to have access to more entrepreneurial information and more entrepreneurial resources through their many social connections, which in effect increases their confidence in entrepreneurship and reinforces their willingness to start a business. Dacin et al. (2010) suggested that to cope with external institutional change, social entrepreneurs must be adept at creatively integrating external resources such as relational, cultural, and institutional resources. Relational resources refer to the use of formal and informal social capital. Subsequently, Meyskens et al. (2010) found that both social network resources and social venture capital can play an important role in the early stages of social enterprise creation.

#### Family factors

Entrepreneurship is closely related to capital, connections, and economic factors, and family is one of the main factors influencing undergraduate students’ willingness to start their own business, with parents’ occupation and capital accumulation affecting the degree of family support for financial self-efficacy, hence stimulate operation of entrepreneurship (Antoncic et al., 2021). Dyer and Handler (1994) believed that the choice of entrepreneurship is more related to their family’s professional background, with students whose parents have experience in entrepreneurship or are entrepreneurs being more likely to have entrepreneurial intentions and to start a business. Entrepreneurial intentions are stronger among students whose parents have had experience in entrepreneurship and whose families are better off financially (Zhou and Li, 2021). The willingness to start a business is stronger for those who have been exposed to family businesses (Tolentino et al., 2014). Hisrich and Ayse Öztürk (1999), who studied female entrepreneurs in developing countries, found that most of them had fathers in business and 22% had access to business capital from their families. Entrepreneurs often profit from family networks even more than from business strategies (Aparicio et al., 2021). At the same time, a slight lack of family support due to partial family responsibilities, such as raising children, and caring for disabled family members, will also lead some people, especially women, to become entrepreneurs (Bögenhold and Fachinger, 2016).

***Hypothesis 2: There is a significant positive effect of acquired characteristics on entrepreneurial intentions***

### Entrepreneurship courses

With the enormous economic potential connected with business venturing, it is no surprise that a broad spectrum of researchers over the past few decades have been driven to figure out whether entrepreneurship can be taught, and if so, how (Arthur et al., 2012). Practicing and potential entrepreneurs gain new knowledge and skills by engaging in management and entrepreneurship-related education, training, and professional development (Antoncic et al., 2007). In the research of university students’ entrepreneurial intention, numerous scholars find that entrepreneurial education plays an extremely essential role. Clouse (1990) empirically analyzed the impact of entrepreneurship education on the entrepreneurial willingness and demonstrated that entrepreneurship education can indeed enhance university students’ entrepreneurial intentions. Brown (2000) found that, in the United States, from an early age, throughout the stages of education, schools will gradually arrange suitable entrepreneurship courses to cultivate students’ entrepreneurial awareness and ability. Entrepreneurship courses can enhance entrepreneurial intentions by improving entrepreneurial skills and competencies, which can lead to entrepreneurial activities, helping educated people to choose the right type of entrepreneurship for them, and giving them direct knowledge about entrepreneurship (Jones and Iredale, 2006). Çera et al. (2021) emphasized the importance of entrepreneurial education, especially since there is a positive correlation between entrepreneurship education and entrepreneurship intention in the context of transition countries. College students who had taken entrepreneurship courses or had experience in entrepreneurship were more significant in the level of entrepreneurial intention (Ye, 2009). Entrepreneurship course design includes the delivery format, quantity, and classification (Zhuo and Ren, 2020). Turker and Selcuk (2009) believed that different types of entrepreneurial education courses in general were predictive of entrepreneurial intentions. Ruskovaara and Pihkala (2014) pointed out the importance of improving teachers’ expertise, encouraging teachers to learn, summarize and provide support in a range of aspects.

***Hypothesis 3: There is a significant positive effect of entrepreneurship courses on entrepreneurial intentions***

### Entrepreneurial practice

Piperopoulos and Dimov (2015) divided entrepreneurship education into entrepreneurship theory and entrepreneurship practice. The former mainly teaches the theoretical knowledge and basic skills of entrepreneurship through classroom teaching, while the latter mainly exercises university students’ ability to cope with difficulties and risks in actual operation through case studies and simulated entrepreneurship. The relationship between entrepreneurship education and students’ experience of entrepreneurial practice has not yet been verified, but research suggests that students who have received entrepreneurship education are more likely to engage in entrepreneurial practice on their initiative. This is because they have a higher level of proactive personality. Entrepreneurs with higher levels of proactive personality spontaneously seek reliable ways to exchange information, build networks, and take positive action as soon as they identify entrepreneurial opportunities in their environment (Bateman and Crant, 1993). The first way for university students to gain information about entrepreneurship through entrepreneurship education is to learn the knowledge and skills of entrepreneurship by attending entrepreneurship courses, but the main way to develop entrepreneurial skills and gain operational management experience is by participating in entrepreneurial practice. Entrepreneurial practice, which focuses on the development of professional skills and the accumulation of entrepreneurial skills and experience, has a more significant impact on entrepreneurial intentions (Wang and Bo, 2016; Ren and Jiang, 2017; Yin, 2019). In other words, after learning appropriate entrepreneurial knowledge in entrepreneurship courses, university students are highly likely to engage in entrepreneurial practice activities in various ways to obtain more effective and high-quality entrepreneurial resources.

***Hypothesis 4: The more entrepreneurial practice among university students has a significant positive effect on entrepreneurial intentions***

### Social support

In the giddy rush to ignite entrepreneurship, a broad range of public and private policy approaches and instruments have been developed and implemented to bestow entrepreneurs with the requisite skills and resources for tax, finance, management, accounting, marketing, human resources, and technological competency (Aly et al., 2021; Audretsch et al., 2021). Obaji and Olugu (2014) proposed that a “favorable business environment, funding policies, and simplification of guidelines to remove bottleneck for emerging entrepreneurs” were popular government entrepreneurial supports. Seymour (2009) describes strong support and willingness to fund entrepreneurship on the part of the United States government and Society, and in fact, a variety of funding sources provide operational support for entrepreneurship. Hart (2009) pointed out that entrepreneurship policy should be systematic and systematically linked to management activities. A range of policies should be formulated according to different levels of economic development, to provide convenient conditions for entrepreneurs and promote the rapid and stable development of enterprise.

To illustrate, China has promoted mass entrepreneurship and innovation since the year 2018, focusing on supporting university graduates in business start-ups. Numerous preferential policies have been inaugurated in support of university students’ efforts at entrepreneurship, such as setting up tax incentives and business incubators and providing business start-up services and training systems (People’s Government of Zhejiang Province, 2015). On top of that, in the field of entrepreneurship, the implication of financial and technical support entrepreneurship can be seen as a resource, the boost confidence and intentions independent of direct assistance. Lin et al. (2017) examined how government entrepreneurial support influences perception, eventually impacting entrepreneurial intentions. However, according to Antoncic et al. (2016), it is clear that marketing self-efficacy may be an important driving force for the establishment of new companies, which is deeply influenced by macroeconomic policies. To illustrate, the effectiveness of policies varies from region, Wei (2019) discovered that college graduates have a low response to the Employment Policy for Graduates in Yunan, a less developed province than Zhejiang.

***Hypothesis 5: There is a significant positive effect of social support on entrepreneurial intentions***

## Data sources and interpretation of variables

### Data sources

The questionnaire used in this paper was based on existing entrepreneurial intentions scales (Fan and Wang, 2006; He, 2006; Gelderen et al., 2008) and was designed using a combination of in-depth semi-structured interviews with several experienced teachers of entrepreneurship education. The first round of pilot testing was distributed to experts and scholars specializing in entrepreneurship education to help refine it, and more than 20 experts and scholars provided valuable comments on the changes. In the second round, the questionnaire was administered to a total of 98 higher education institutions across China to test the reliability and validity of the questions. In the third round, based on the suggestions of experts and scholars, we held several meetings to discuss and revise the questionnaire item by item and formed the final questionnaire.

The survey was taken from undergraduate and college students who had received innovation and entrepreneurship education in 1231 universities in 31 provinces (autonomous regions and municipalities directly under the central government) in mainland China, and a total of 87,914 questionnaires were collected. After excluding 5,305 invalid questionnaires due to short response time or invalid school names, 82,609 valid questionnaires remained, accounting for 93.97% of the total. Among them, 28,610 were male students and 53,999 were female students, 53,015 were non-urban household registration and 29,594 were urban household registration, 29,445 were only children and 53,164 were non-only children, 76,937 were Han Chinese and 5,672 were ethnic minorities (as shown in Table 1).

**TABLE 1:** Table of data sources.

Projects	Category	Number of frequencies/persons	Proportion (%)
Gender	Male	28,610	34.6
	Female	53,999	67.6
Type of household registration	Rural	53,015	64.2
	Towns	29,594	35.8
One-child	Yes	29,445	35.6
	No	53,164	64.4
Ethnicity	Han Chinese	76,937	93.1
	Minorities	5,672	6.9

### Research tools and variable setting

In this study, whether college students have the intention to start a business is taken as the dependent variable, and the factors influencing college students’ intention to start a business are divided into five independent variables: innate endowment, acquired characteristics, entrepreneurial curriculum, entrepreneurial practice, and social support (see Table 2). Among them, innate endowment includes four indicators: gender, ethnicity, and whether they are only children; acquired characteristics include five indicators: type of household registration, discipline, social resources, their own skills, and family factors; entrepreneurial curriculum includes teachers with entrepreneurial experience, teachers with rich experience in professional education and teaching, entrepreneurial courses offered, entrepreneurial course content closely integrated with their own professional knowledge, and entrepreneurial courses integrated with cutting-edge trends Entrepreneurial practice includes instructors inside and outside the university for entrepreneurial practice, support from a special entrepreneurial fund for entrepreneurial practice, an independent university business park for entrepreneurial practice, a special off-campus practice base for entrepreneurial practice, and a high degree of integration between entrepreneurial practice projects and professional studies; social support includes the degree of policy support, the degree of support from the university, the state’s tax exemption for college students starting their own businesses, the local government’s simplification of the application process for college students’ business registration The social support includes the degree of policy support, the degree of support from the school, the state’s reduction of enterprise tax for college students, the local government’s simplification of college students’ enterprise registration application process, the school’s provision of start-up fund as an interest-free loan, and the society’s provision of free training to guide entrepreneurship. Except for the independent variables of individual innate endowment and acquired characteristics, which were measured categorically and subject categories were assigned numerically, all other items were scored on a 5-point Likert scale (5: strongly agree/very important; 4: relatively agree/more important; 3: average; 2: relatively disagree/less important; 1: strongly disagree/very unimportant) according to the university students’ evaluation of their importance. For the dependent variable, willingness to start a business was coded as “1” and unwillingness as “0.”

**TABLE 2:** Description of variables and descriptive results statistics.

Variables	Measurement items	Explanation of variables	Average	Standard deviation
Dependent variable	Willingness to start your own business after graduation	1: willing;0 :not willing	0.900	0.285
Independent variable				
Independent variable factor 1: Innate endowment	Gender	1:male; 0:female	1.650	0.476
	Ethnicity	1: Han Chinese; 0: ethnic minority	1.071	0.253
	One-child	1:only child; 0:non-only child	1.641	0.479
Independent variable factor 2: Acquired characteristics	Type of household registration	1:town; 0:non-town	1.642	0.479
	Academic discipline	1: philosophy 2: economics 3: law 4: education 5: literature 6: history 7: science 8: engineering 9: agronomy 10: medicine 11: military 12: management 13: art	7.853	3.284
	Social resources	5: strongly agree/very important 4: somewhat agree/more important 3: generally 2: relatively disagree/relatively unimportant 1: strongly disagree/very unimportant	2.364	1.069
	Skill		2.632	0.989
	Family factors		1.781	0.414
Independent variable factor 3: Entrepreneurship courses	Teachers have experience in entrepreneurship	5: strongly agree/very important 4: somewhat agree/more important 3: generally 2: relatively disagree/relatively unimportant 1: strongly disagree/very unimportant	3.331	0.990
	Teachers have extensive experience in teaching entrepreneurship education		3.460	0.984
	Number of entrepreneurship courses offered		1.801	0.562
	The content of the entrepreneurship course is closely integrated with their professional knowledge		3.253	1.015
	Entrepreneurship course content is closely aligned with current trends		3.443	0.966
Independent variable factor 4: Entrepreneurial practice	Entrepreneurial practice with mentors inside and outside the comb	5: strongly agree/very important 4: somewhat agree/more important 3: generally 2: relatively disagree/relatively unimportant 1: strongly disagree/very unimportant	3.602	0.936
	Special entrepreneurship fund for entrepreneurial practice		3.531	0.956
	There is an independent entrepreneurial park for entrepreneurial practice		3.552	0.987
	There is a special off-campus practice base for entrepreneurial practice		3.443	0.978
	High degree of integration of entrepreneurial practice projects with professional studies		3.464	0.956
Independent variable factor 5: Social support	Level of policy support	5: strongly agree/very important 4: somewhat agree/more important 3: generally 2: relatively disagree/relatively unimportant 1: strongly disagree/very unimportant	3.654	0.827
	Degree in school support		3.506	0.857
	State tax relief for college students starting their businesses		3.661	0.907
	Local government simplifies the application process for college student business registration		3.631	0.908
	Interest-free loans from the university for business start-up funds		3.603	0.934
	Free training from the society to start a business		3.571	0.951

### Statistical methods

The dependent variable in the study is dichotomous and should be subjected to binary Logistic regression analysis. In the regression model, the dependent variable is set to be P, taking values of 0 and 1 and obeying a binomial distribution. The n independent variables affecting P are denoted as *X*1, *X*2,…, and *X*n. Based on this set-up model, the analysis was conducted using SPSS 21.0.


$$L\,o\,g\,i\,t\,\left(P\right)=\beta_{0}+\sum_{i=1}^{n}\beta_{i}\,X_{i}$$

where *P* is the willingness to engage in self-employment after graduation, with a value of 1 indicating willingness and 0 indicating unwillingness. *X*1, *X*2, *X*3, *X*4, and *X*5 are innate endowment, acquired characteristics, entrepreneurship courses, entrepreneurial practice, and social support, respectively. β_0_ is a constant and *β_*i*_* is the bias regression coefficient corresponding to *X*i (*i* = 1, 2,…, *n*). To avoid possible multicollinearity with more independent variables and to reflect more clearly the differences in the influence of different levels of independent variables on the intention to start one’s own business after graduation, stepwise regression analysis was used to first examine the influence of the factors of innate endowment, acquired characteristics, entrepreneurship courses, entrepreneurship practice and social support on the dependent variable one by one, and then comprehensively examine the influence of all independent variables on the dependent variable.

## Results and analysis

### Descriptive statistics of influencing factors

Workforce diversity, technological innovation, and globalization trends have changed traditional organizational structures and work environments and made entrepreneurship a popular career choice for university students. However, despite the prevalence of entrepreneurial intentions, only a minority have transformed them into substantive entrepreneurial behavior and adopted it as a career. This study, therefore, examines the influence of individual innate endowments, acquired characteristics, entrepreneurship courses, entrepreneurial practices, and social support factors on entrepreneurial intentions.

Before this, descriptive statistics are provided for each factor to provide a clearer picture of the current state of the factors influencing entrepreneurship among university students. The entrepreneurship course factor includes entrepreneurial experience, experience in teaching entrepreneurship, the number of courses offered, how closely the content is aligned with professional knowledge, and how closely the content is aligned with current trends. As can be seen from the above table, the mean value of the “number of entrepreneurship courses offered” is 1.801, the lowest compared to other factors, indicating that there is a greater demand for entrepreneurship courses among university students, but the actual situation is far from satisfactory. On the other hand, the mean values for entrepreneurship experience, experience in teaching entrepreneurship education, how closely the content is integrated with professional knowledge, and how closely the content is integrated with current trends were 3.331, 3.460, 3.253, and 3.443, respectively, indicating a higher level of agreement among students. The comparison shows that the number of entrepreneurship courses offered has the lowest mean score among all the factors of entrepreneurship courses, which indicates that university students have the lowest agreement with the number of entrepreneurship courses offered.

The entrepreneurial practice factor is also one of the most important factors, including mentors, special entrepreneurship fund support, integrated entrepreneurial practice services, independent college students’ entrepreneurship park, a special off-campus practice base, and the degree of integration of entrepreneurial practice projects with professional studies, covering a wide range of factors. The average score of all the factors in the entrepreneurial practice factor is above 3, with the factor of “having an external mentor for entrepreneurial practice” having the highest average score of 3.602; the average scores of other factors in the entrepreneurial practice factor fluctuate less, with the average scores of “special entrepreneurship fund support,” “independent entrepreneurship park for college students,” “special off-campus practice base,” and “integration of entrepreneurial practice projects with professional studies.” The mean values of the other factors of the entrepreneurial practice factor fluctuate less, with the mean values of 3.531, 3.552, 3.443, and 3.464, respectively, for the factors of support from a special entrepreneurship fund, independent entrepreneurship park, special off-campus practice base, and integration of entrepreneurial practice projects with professional studies, thus the factor of “having a special off-campus practice base for entrepreneurial practice” has the lowest mean value, which shows that there is some room for improvement in the construction of off-campus practice bases.

The social support factor is a factor that cannot be ignored. The social support factor consists of the degree of policy support, the degree of school support, the state’s tax exemption for college students to start their own business, the local government’s simplification of the application process for college students’ business registration, the school’s provision of an interest-free loan for the start-up fund of the business, and the free training for the business. The mean scores of the factors of policy support, school support, state tax exemption for students starting their businesses, local government simplifying the application process for students’ business registration, school providing interest-free loans for business start-up funds, and free training for business start-up are 3.654, 3.506, 3.661, 3.631, 3.603, and 3.571, respectively, which show that the mean scores of each subdivision of the social support factor The mean scores for each of the social support factors are all above the medium level.

In summary, the mean scores for each of the entrepreneurship courses, entrepreneurship practice, and social support factors are mostly above average, while the lowest scores are for the number of entrepreneurship courses offered by university students, who show a greater demand for entrepreneurship courses while focusing on the practicality of entrepreneurship policies.

### Statistical analysis of the model

Six models were developed to test the effects of innate endowments, acquired characteristics, entrepreneurial curriculum, entrepreneurial practices, and social support factors on the dependent variables, with the following results.

#### Innate endowment

The results of the analysis of Model 1 (Table 3) show that the variables of gender, ethnicity, and whether they are only children of university students passed the significance test, with the willingness of female students to start a business after graduation being 0.347 times that of male students, and the willingness of female students to start a business after graduation being relatively weak. Compared to ethnic minority college students, Han Chinese college students had the highest intention to start their own business after graduation, 1.437 times higher than ethnic minority college students. Compared to non-only children, only children were 1.135 times more likely to start a business after graduation. Model 6, after fully examining the effects of all the independent variables on college students’ willingness to start a business after graduation, found that the effects of all the independent variables remained significant.

**TABLE 3:** Results of regression analysis of innate endowment factors on the dependent variable.

Variable		Model 1		Model 6	
		B	Exp (B)	B	Exp (B)
Innate endowment	Gender	−1.059***	0.347	−0.906***	0.404
	Ethnicity	0.363***	1.437	0.365***	1.440
	One-child	0.126***	1.135	0.137***	1.145

*p < 0.05, **p < 0.01, ***p < 0.001.

#### Acquired characteristics

The results of Model 2 (Table 4) show that the type of hukou, academic discipline, social resources, their skills, and family factors all passed the significance test, and the willingness to start a business after graduation was 1.357 times higher among students with urban hukou than those with non-urban hukou. In addition, the willingness to start a business after graduation increases by 1.023 times when the possession of social resources increases by 1 level (5 levels in total, the same below); the willingness to start a business after graduation increases by 1.748 times when the level of their skills increases by 1 level; and the willingness to start a business after graduation decreases by 0.545 times when the family factor decreases by 1 level. The above factors are considered together in model 6, so the variables are still highly significant.

**TABLE 4:** Results of regression analysis of acquired characteristics factors on the dependent variable.

Variable		Model 2		Model 6	
		B	Exp (B)	B	Exp (B)
Acquired characteristics	Type of household registration	0.306***	1.357	0.240***	1.271
	Academic discipline	0.027***	1.027	0.023***	1.024
	Social resources	0.022***	1.023	0.043***	1.044
	Skill	0.558***	1.748	0.530***	1.699
	Family factors	−0.607***	0.545	−0.604***	0.546

*p < 0.05, **p < 0.01, ***p < 0.001.

#### Entrepreneurship courses

The results of Model 5 analysis showed that all independent variables passed the significance test (Table 5). Entrepreneurship courses are an important influencing factor on whether or not college students start their own business after graduation. 1 level increase in teachers’ experience in entrepreneurship increases college students’ willingness to start their own business after graduation by 1.102 times; 1 level decrease in teachers’ experience in teaching entrepreneurship education decreases college students’ willingness to start their own business after graduation by 0.924 times; 1 level increase in the number of entrepreneurship courses started increases college students’ willingness to start their own business after graduation by The willingness to start a business after graduation increases by 1.173 times for each increase in the number of entrepreneurship courses started, by 1.098 times for each increase in the number of entrepreneurship courses combined with their professional knowledge, and by 1.040 times for each increase in the number of entrepreneurship courses combined with current trends. When the effects of all independent variables on the dependent variable were examined (Model 6), the effects of teachers having extensive experience in teaching entrepreneurship education, the number of entrepreneurship courses offered, and the close integration of entrepreneurship course content with their professional knowledge remained significant.

**TABLE 5:** Results of regression analysis of entrepreneurship curriculum factors on dependent variables.

Variable		Model 3		Model 6	
		B	Exp (B)	B	Exp (B)
Entrepreneurship courses	Teachers with entrepreneurial experience	0.097***	1.102	–0.011	0.989
	Teachers with extensive experience in teaching entrepreneurship education	−0.079***	0.924	−0.070***	0.932
	Number of entrepreneurship courses offered	0.159***	1.173	0.101***	1.106
	The content of the entrepreneurship course is closely integrated with your professional knowledge	0.093***	1.098	−0.065***	0.937
	The content of the entrepreneurship course is closely aligned with current trends	0.039***	1.040	0.031	1.032

*p < 0.05, **p < 0.01, ***p < 0.001.

#### Entrepreneurial practice

The results of model 4 analysis showed that entrepreneurial practice supported by a special entrepreneurship fund, entrepreneurial practice with a special off-campus practice base, and entrepreneurial practice projects with a high degree of integration with professional studies passed the significance test. For each level of support from a special entrepreneurship fund, the willingness to start a business after graduation decreases by 0.917 times; for each level of support from a special off-campus practice base, the willingness to start a business after graduation increases by 1.098 times; for each level of integration between entrepreneurship projects and professional studies, the willingness to start a business after graduation increases by 1.079 times. When the effects of all independent variables on the dependent variables are examined (model 6), the effects of having on-campus and off-campus instructors for entrepreneurial practice, having an independent entrepreneurial park for college students, having a special off-campus practice base for entrepreneurial practice, and having a high degree of integration between entrepreneurial practice projects and professional studies are significant, among which having a special off-campus practice base for entrepreneurial practice and having a high degree of integration between entrepreneurial practice projects and professional studies are significant in both model 4 and model 6 This further illustrates the strength of their influence on college students’ intention to start a business after graduation (see Table 6).

**TABLE 6:** Results of regression analysis of entrepreneurial practice factors on the dependent variable.

Variable		Model 4		Model 6	
		B	Exp (B)	B	Exp (B)
Entrepreneurial practice	Entrepreneurial practice with on and off-campus mentors	0.032	1.032	0.098*	1.103
	Entrepreneurial practice is supported by a dedicated start-up fund	−0.086***	0.917	0.018	1.018
	There is an independent student business park for entrepreneurial practice	0.035	1.035	0.126***	1.134
	Dedicated off-campus practice base for entrepreneurial practice	0.094***	1.098	0.131***	1.140
	A high degree of integration of practical entrepreneurship projects with professional studies	0.076***	1.079	0.088*	1.091

*p < 0.05, **p < 0.01, ***p < 0.001.

#### Social support

The results of Model 5 analysis showed that the level of policy support, the level of school support, and the start-up fund (interest-free loan) provided by the school to start a business passed the significance test. For each level of policy support, the willingness of college students to start a business after graduation increased by 1.461 times; for each level of school support, the willingness of college students to start a business after graduation increased by 1.071 times; for each level of school start-up fund (interest-free loan), the willingness of college students to start a business after graduation decreased by 0.840 times. When the effects of all independent variables on the dependent variable were examined (Model 6), the effects of all independent variables were significant. Among them, state tax relief for college students starting their businesses, local government simplifying the application process for college students’ business registration, and society providing free training to guide entrepreneurship were significant in Model 6, indicating that the effects of these variables were magnified when college students considered all elements collectively (see Table 7).

**TABLE 7:** Results of the regression analysis of social support factors on the dependent variable.

Variable		Model 5		Model 6	
		B	Exp (B)	B	Exp (B)
Social support	Level of policy support	0.379***	1.461	0.515***	1.674
	Level of school support	0.069***	1.071	−0.547***	0.579
	State tax relief for university students starting their businesses	–0.053	0.949	−0.076*	0.927
	Local governments simplify the application process for university student business registration	–0.016	0.984	−0.088*	0.916
	The university provides a start-up fund (interest-free loan) for starting a business	−0.174***	0.840	−0.169***	0.844
	Free training provided by the community to guide entrepreneurship	–0.019	0.981	−0.089***	0.915

*p < 0.05, **p < 0.01, ***p < 0.001.

## Conclusion based on empirical results

The analysis leads us to the following conclusion. The traditional Chinese culture of son-ship is not evident in entrepreneurship, meaning that the act of entrepreneurship presents a paradoxical pull of openness and closure in China. The number of schools that offer entrepreneurship courses bears a significant impact on entrepreneurial intentions (Postigo and Tamborini, 2004; Trivedi, 2016). The more courses that are opened, the higher the entrepreneurial intentions will be. Current entrepreneurship courses are divided into three categories: cognitive courses based on basic entrepreneurial theory, experimental semi-practical courses based on competitions and supervised research projects, and courses based on a variety of technological and scientific research projects. In terms of quantity and format, China’s entrepreneurship courses should be able to meet the needs of students, but in contrast to the requirements for teachers, we can assume that the content of entrepreneurship courses and the subjects taught are far from meeting the needs of students. Entrepreneurship is now seen as a means to quality employment in China, but there is still no consensus on what entrepreneurial skills to have and what kind of entrepreneurship education to provide to achieve the goal of quality employment.

## Discussion

We suggest that five aspects–innate endowment, acquired characteristics, entrepreneurial courses, entrepreneurial practice, and social support–have a significant impact on entrepreneurial intentions and consider these five factors to be key factors in making entrepreneurial decisions. The entrepreneurship curriculum should be gradually transformed from the current one based on school teachers to one implemented jointly by teachers + business people + alumni, and external teachers from the school should be given the same recognition in terms of title assessment. The content of the curriculum should be dynamic and reflect changes in the industry and social trends. In terms of policy implementation, the state should provide precise evaluation and support for science and technology parks and business incubators in schools, taking the evaluation of “double first-class” or “double universities” as an example, and introduce specific policies to support them. Optimize the protective environment for entrepreneurial activities and increase students’ motivation to start their businesses. Entrepreneurship education is still at the level of theoretical discussion, and promoting more entrepreneurship among university students requires more support from the curriculum and teachers’ experience in school, but Chinese society is still relatively conservative, and “risky” entrepreneurship instead of a stable organization. However, Chinese society is still relatively conservative and most people have reservations about ‘risky’ entrepreneurship as a substitute for stable organizational ‘employment’ and it is recommended that students who fail to start their own business be supported in terms of finance and skills training.

The theoretical contribution of this study is the identification of five major elements, compared to those who focus more on entrepreneurship education, we find that social support seems to be valued, which can reduce entrepreneurial risk and support initial capital. This is contrary to the findings of Krueger (2007) and Chen and Shi (2020) “entrepreneurship is a motivational behavior” and “the business opportunity to start a business is rather determined by the entrepreneur’s willingness to start a business.” More students realize that starting a business is not easy and although there are more and more policies at a national level to encourage entrepreneurship rather than looking for a certain position in an organization, students are getting more and more information from the internet and find that there are far more failures than success stories. They try to gain entrepreneurial knowledge and skills and analyze market trends through entrepreneurship education, rather than starting a business unless there is a steady stream of policy support and opportunities.

In terms of practical contributions, this study has two implications for policymakers and researchers in entrepreneurship education. In terms of the independent variables, this study takes into account respondents’ own factors and environmental influences, particularly gender and household registration among the individual characteristics, which are key factors influencing entrepreneurial intentions, and are characteristic of local Chinese practice research. The study is also relevant to the implementation of the Common Wealth in China, and it is important to consider the “urban-rural dichotomy” and the advancement of women in China, to provide methodologies and ideas for future support policies and entrepreneurial projects for key groups. In terms of the entrepreneurship curriculum, it is clear that entrepreneurship courses have an impact on students’ willingness to start their businesses. However, in our analysis, we found that the current entrepreneurship curriculum still needs to be more closely aligned with students’ majors and that there is a lack of generalist courses around business plan collaboration and communication. At the same time, entrepreneurship guidance from schools is often not sustainable after graduation, which makes students less satisfied with the content of entrepreneurship education. In future practical research, the extension of entrepreneurship courses from school to training should be established, especially for students who are willing to start a business and participate in entrepreneurial competitions and project incubation needs to be ongoing education.

## Limitations and future research recommendations

Due to the large number and diverse types of institutions involved in this study, including higher education institutions implementing both general and vocational education, the time frame is long and the latest policy requirements cannot be absorbed in the process of analyzing the data on time, resulting in a lack of dynamic and real-time analysis results, which needs to be improved in future studies.

The study is not sufficiently typological, and the study is general but lacks specific analysis for particular types of institutions. For example, the newly promulgated Law of the People’s Republic of China on Vocational Education and the proposal to build a skill-based society have led to an increased interest in vocational education in general, and whether the entrepreneurial intentions and behaviors of students in vocational institutions have their unique characteristics is something that future research needs to focus on.

In the process of constructing the indicators, the study of students’ entrepreneurial intentions and their career development trajectories 1, 3, and 5 years after graduation are not sufficiently integrated.

## Data availability statement

The original contributions presented in this study are included in the article/supplementary material, further inquiries can be directed to the corresponding author.

## Author contributions

YL and CC contributed to the research topic and framework. YL designed the field survey and analyzed the data. RW wrote the manuscript and reviewed the literature. CC wrote the strategy and edited the manuscript. All authors contributed to the article and approved the submitted version.

## Funding

This article was funded by Zhejiang Provincial Philosophy and Social Science Planning Project (20JDZD044 and 21NDJC308YBM) and Zhejiang Provincial Education Science Planning Project (2022SCG208).
